# From Natural Resources Evaluation to Spatial Epidemiology: 25 Years in the Making

**DOI:** 10.1007/s11004-020-09886-x

**Published:** 2020-08-28

**Authors:** P. Goovaerts

**Affiliations:** 2 BioMedware, 167 Little Lake Drive, Ann Arbor, MI 48106, USA.

**Keywords:** Poisson kriging, medical geography, environment, cancer

## Abstract

When in the winter of 1994, under the supervision of my post-doc adviser André Journel, I started writing “*Geostatistics for Natural Resources Evaluation*” in the bedroom of a tiny Palo Alto apartment, little did I know that 25 years later I would be conducting NIH-funded research on medical geostatistics from a lakefront office nestled in the Irish Hills of Michigan. The professional and personal path that led me to trade the mapping of heavy metal concentrations in the topsoil of the Swiss Jura for the geostatistical analysis of cancer data was anything but planned, yet André’s help and guidance were instrumental early on. Looking back, shifting scientific interest from the characterization of contaminated sites to human health made sense as the field of epidemiology is increasingly concerned with the concept of exposome, which comprises all environmental exposures (e.g., air, soil, drinking water) that a person experiences from conception throughout the life course. Although both environmental and epidemiological data exhibit space-time variability, the latter has specific characteristics that required the adaptation of traditional geostatistical tools, such as semivariogram and kriging. Challenges include: (i) the heteroscedasticity of disease rate data (i.e., larger uncertainty of disease rates computed from small populations), (ii) their uneven spatial support (e.g., rates recorded for administrative units of different size and shape), and (iii) the limitations of Euclidean metrics to embody proximity when dealing with data that pertain to human mobility. Most of these challenges were addressed by borrowing concepts developed in adjacent fields, stressing the value of interdisciplinary research and intellectual curiosity, something I learned as a fresh PhD in agronomical sciences joining André’s research group at the Stanford Center for Reservoir Forecasting in the early nineties.

## 1. Introduction: my Time at Stanford

“*Pierre, I know you can do it… I want a book written by you, in your style… A book is like a baby, it needs only one father… and I plan to write my own book during my sabbatical.*” I vividly remember this pep talk given by André Journel in February 1994, as I was ready to throw in the towel on writing a book and had suggested we collaborate on that daunting project. This conversation, or rebuttal, took place in his office located at the top floor of the freshly built Green Earth Sciences building, far away from other faculty members as he enjoyed keeping a close eye on his graduate students. This was one of many instances where André would boost my confidence and encourage one of his students to push their limits and aim high.

Eight months earlier, we had started discussing the book project during a flight to Montreal on our way to attend the 1993 International Forum titled “Geostatistics for the Next Century”. André was sitting next to me, reviewing student papers as he likes staying busy. (Incidentally, he forgot the annotated copies in the seat pocket and never recovered them since the plane had been cleaned by the time he realized his oversight.) He casually mentioned his plan to edit a book series for Oxford University Press and suggested I write a short book on multivariate geostatistics. I was flattered by this mark of confidence as my visit to Stanford had not started under the best auspices. Indeed, in April 1992, I embarked on my first air travel ever, a 14-hour transatlantic flight from Brussels to San Francisco, to visit André in preparation for my forthcoming post-doctoral stay at the Stanford Center for Reservoir Forecasting (SCRF). Jittered by my encounter with the author of *Mining Geostatistics*, I spilled my coffee on André’s meticulous desk, before giving him the wrong country code as he offered to dial my home phone number for a quick reassuring call to family. This nightmarish introduction was followed two days later by my first seminar in English, which had been scheduled late afternoon in a windowless room of the Mitchell Earth Sciences Building. Battling the 9-hour jet lag as my biological clock was still set on 1 a.m., my carefully prepared presentation quickly melted into an excruciating 1.5-hour messy marathon. I was being bombarded by questions and requests for graphs that a frenetic search through a big stack of transparencies could not fulfill. As André was giving me a ride to the airport at the end of this two-week visit, he expressed second thoughts about his initial offer to fund my second year as a post-doc. Things, however, turned around when a research paper on indicator (co)kriging of categorical variables I had prepared for the 1993 SCRF meeting was surprisingly well received by André who invited me to present the results in Montreal as a reward.

Most of my book was written at home; the desk was a garden table I had bought for $5 at a garage sale. There were no laptops in the early nineties, and the only computers we had access to were bulky Unix workstations located on campus. Thus, the only option was to draft the manuscript by hand before going to the office to type it. André had a similar *modus operandi*, and one day in December 1993, he asked me to join him on a trip to the Stanford bookstore. There, he taught me how to shop for all the items indispensable to write that book: (i) a #2 pencil (in his words, “Don’t be arrogant and think you will never make a mistake; hence, always use a pencil”), (ii) a rubber eraser, (iii) a quadrille-ruled notebook, and (iv) last, but not least, some adhesive tape to cut and paste paragraphs. Equipped with all these tools and relying on an outline I had drafted during another transatlantic flight, I was ready!

**Figure 1.**
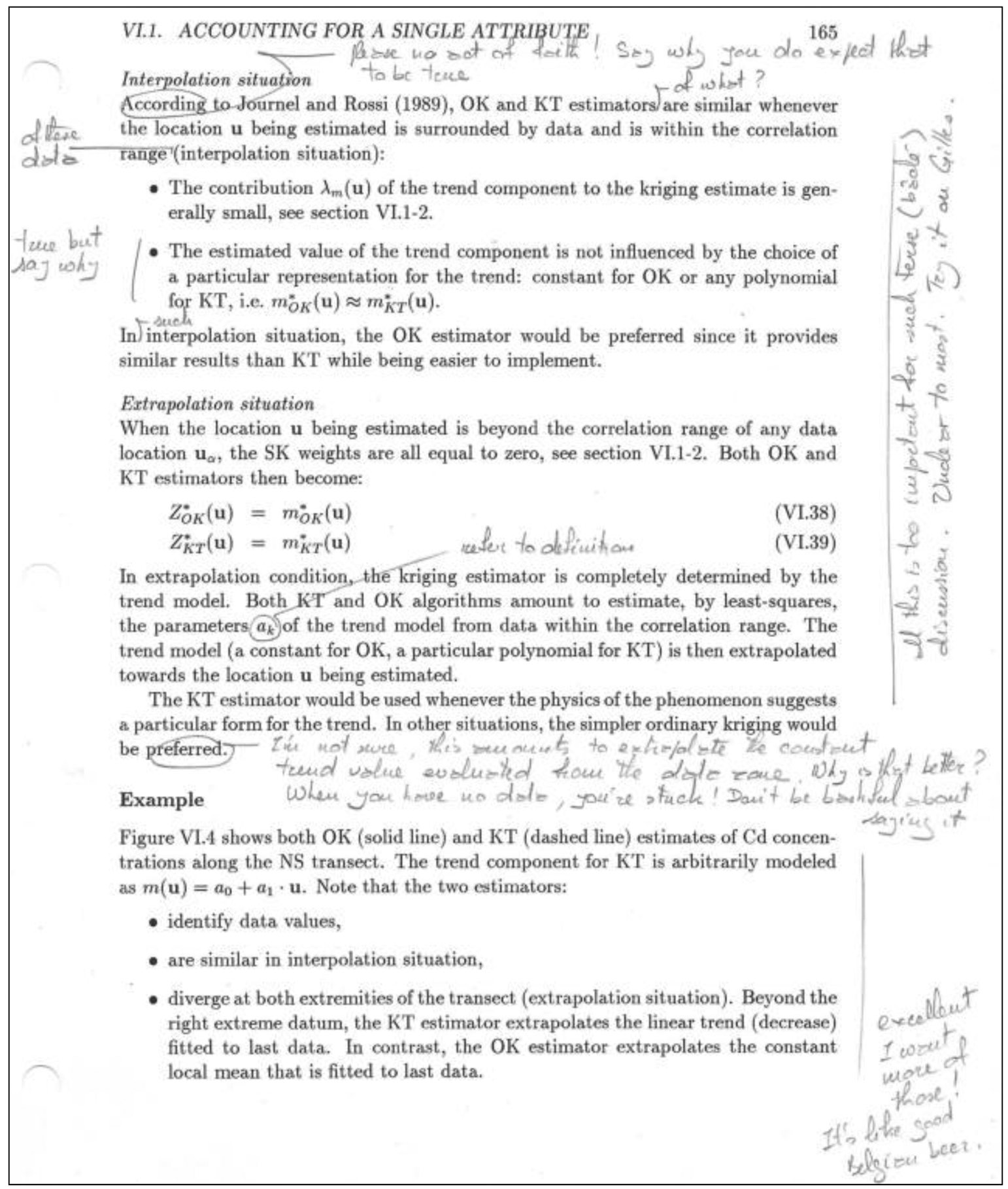
The first draft of the book “*Geostatistics for Natural Resources Evaluation*” with André Journel’s annotations in pencil

The book ended up being a success, thanks in large part to André’s tireless revisions and rigorous comments during the 18-month gestation. He emphasized the importance of making the text crisp and readable, getting rid of all redundancies, unnecessary words and expressions, what he likes to call the *Baroque*, in reference to a highly ornate and often extravagant style of architecture that flourished in Europe in the 17^th^-18^th^ century. My draft would be heavily annotated in pencil (Fig. 1) and include some of André’s favorite expressions, such as, *Prends ton fer forgeron*! (*Take your iron blacksmith!* in reference to the need to revise the text). Even during his sabbatical in France, André would request that I regularly snail mail chapters of my book for review so he could monitor my progress from his little apartment located in the Latin Quarter, Paris. After reading my chapter on kriging, he decided there was no need for him to write another book on geostatistics, which, he said should be done by a younger person. Instead, a better use of his time would be enjoying his sabbatical, jogging through the Jardin du Luxembourg and feasting on the French cuisine every other day.

Half of the book *Geostatistics for Natural Resources Evaluation* (Goovaerts 1997) is devoted to the modeling of local and spatial uncertainty, two topics I knew little about when I started my post-doctoral fellowship in January 1993. Upon my arrival, André strongly recommended that I take a clean break from the research on multivariate geostatistics I had conducted during my Ph.D. to start learning new skills and methods. I was allowed to audit his class on geostatistics under the condition that I completed all assignments, which entailed analyzing diverse datasets using the recently developed Gslib code (Deutsch and Journel 1998). Learning about the theory and application of indicator kriging and stochastic simulation not only were instrumental in writing several chapters of my book, but later proved invaluable for the analysis of health data and supported the development of new approach to statistical testing of spatial data (see Sect. 7).

In December 1994, I said good bye to the Farm and flew back to Belgium with the hope that my productive time at Stanford would suffice to secure a faculty position at my alma mater, The Catholic University of Louvain-la-Neuve. Two years later, as I was still waiting for the green light from my University, I flew to Australia to attend the 1996 Geostat conference in Wollongong. During the tour of an Australian rainforest, André gave me another pep talk, stating that Belgium was too small for me, and it was time I return to the US, specifically at the University of Michigan where a tenure-track faculty position was open for a geostatistician in the department of Civil and Environmental Engineering. Thanks to Andre’s strong letter of recommendation and a few tips on social interactions, such as, “Pierre, don’t look at your shoes during the faculty interviews,” I nailed my first job interview and was back in the States.

André was notably absent from the latest episode in my professional career, namely my growing interest in the field of environmental epidemiology, which culminated with my appointment as chief scientist of the R&D company, BioMedware, Inc. in the Fall of 2002. Four years later, I shared this new interest with André and his group during a seminar “Modeling the Impact of the Environment on Human Health: How can Geostatistics and Space-Time Information Systems help you?” André then recognized the challenges of analyzing the space-time distribution of health outcomes, as they reflect the impact of a myriad of factors (environmental, socio-economic, behavioral, demographic, and genetic) that operate and interact at different scales. In this paper, I explain the challenges faced on my journey to tailor the methods learned in the nineties at SCRF to the analysis of health data. The solution itself is not always described in detail since most of this work has already been published in this journal. The focus is more on how these ideas came to fruition, sometimes randomly, often through interdisciplinary research, and always because of intellectual curiosity.

## 2. My Naïve Attempt to Apply Geostatistics to Cancer Data

**Figure 2.**
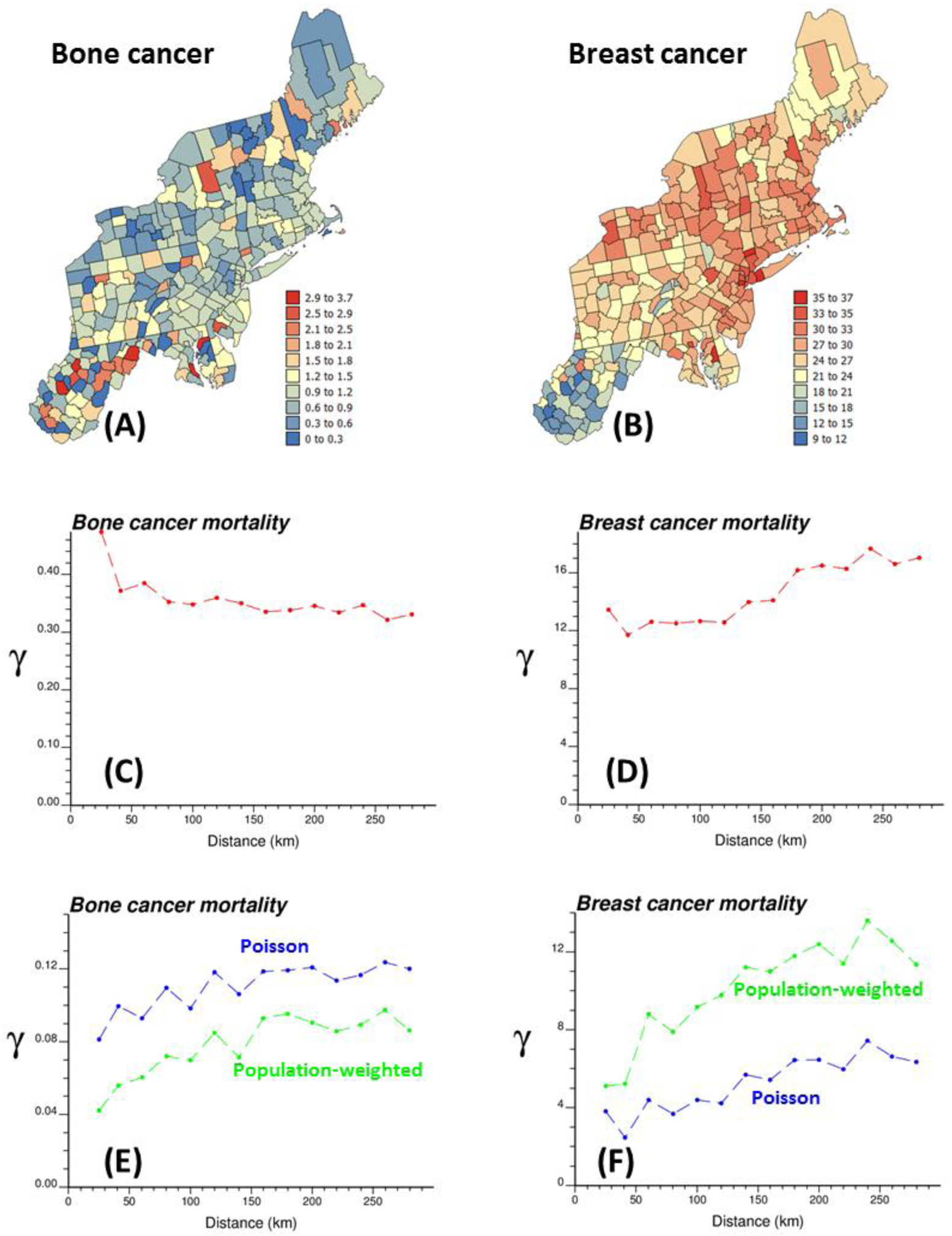
Maps of bone and breast cancer mortality rates (units = number of deaths per 100,000 habitants) recorded during the 1950–1969 period for 295 counties of 12 New England States (A,B). Bottom graphs show the semivariogram of mortality rates computed using: the traditional (unweighted) estimator (C,D), and the population-weighted and Poisson estimators (E,F)

Most geostatisticians remember their first experimental semivariogram and the model they fitted, often painstakingly. In my case, I was analyzing the spatial pattern of topsoil pH data I had collected as an undergraduate student in farm fields and forests of the Fagne de Chimay (Goovaerts 1998). My first geostatistical analysis of cancer data was equally memorable. At the time, I was still on the faculty at the University of Michigan and had to rely on BioMedware staff to assemble a cancer dataset for testing. One employee quickly accessed the *Atlas of Cancer Mortality for U.S. Counties* and downloaded mortality rates for 295 counties of 12 New England States (1950–1969 period); he selected the first four cancers in alphabetical order: bladder, bone, brain, and breast, a convenient choice I suppose; see maps of bone and breast cancer mortality rates in Fig. 2A–B.

Using the traditional estimator available in most software, the experimental semivariograms turned out to be quite erratic, in particular for bone cancer (Fig. 2C). This was my first encounter with the “small number problem” (Waller and Gotway 2004): mortality rates computed from sparsely populated areas or for rare diseases (e.g., brain and bone cancers are less prevalent than breast and bladder cancers) tend to be less reliable, leading to noisy semivariograms. One can view this noise as the result of measurement errors that prevent the accurate quantification of the underlying risk of dying from cancer, with the caveat that the variance of measurement errors is non-stationary as the population size varies spatially. In addition, the aggregation of health data within administrative units of widely different sizes and shapes results in a spatially heterogeneous regularization of the semivariogram. Clearly, the naïve application of traditional geostatistical tools was not the way to go.

## 3. Binomial Cokriging: a First Step in the Right Direction

The first initiative to tailor geostatistical tools to the analysis of disease rates must be credited to Christian Lajaunie (1991) from the Center of Geostatistics in Fontainebleau, France. He developed an approach that accounts for spatial heterogeneity in the population of children to remove the noise from the semivariogram of observed childhood mortality rates. For a given number *m* of geographical units *v*_*i*_ (e.g., counties, census tracts), denote the observed disease rates (areal data) as z(*v*_*i*_)=y_*i*_/n(*v*_*i*_), where y_*i*_ is the number of recorded cases and n(*v*_*i*_) is the size of the population at risk. These units are referenced geographically by their population-weighted centroids **u**_i_=(x_i_,y_i_). The semivariogram of the “risk of developing cancer”, ${\hat{\gamma }}_{R}(h)$, is derived from the semivariogram of observed mortality rates, ${\hat{\gamma }}_{Z}(h)$, as

(1) $${\hat{\gamma }}_{R}(\text{h})={\hat{\gamma }}_{Z}(\text{h})-\frac{1}{2}\left\{\bar{z}(1-\bar{z})-{\hat{\sigma }}_{R}^{2}\right\}\left\{\frac{1}{N(\text{h})}\overset{N(\text{h})}{\underset{i,j}{\sum }}\frac{n\left(v_{i}\right)+n\left(v_{j}\right)}{n\left(v_{i}\right)\times n\left(v_{j}\right)}\right\},\;\text{with}$$

(2) $${\hat{\gamma }}_{Z}(h)=\frac{1}{2N(h)}\overset{N(h)}{\underset{i,j}{\sum }}{\left[z\left(v_{i}\right)-z\left(v_{j}\right)\right]}^{2},$$

where $\bar{z}$ is the population-weighted average of rates, N(**h**) is the number of pairs of areas (*v*_*i*_,*v*_*j*_) whose population-weighted centroids (**u**_i_, **u**_j_) are separated by the vector **h**. An iterative procedure is used to estimate the variance of the risk ${\hat{\sigma }}_{R}^{2}$ which is a priori unknown, see Oliver et al. (1998) for a more detailed description. Application of formula (1) to New England cancer data led to negative values for the experimental semivariogram of the risk (Goovaerts 2005a), a disconcerting feature that was observed on various datasets with different geographies and population sizes. According to simulation studies, this problem is caused by the fact that all developments underlying the derivation of Eq. (1) are based on the modeling of the error term as a binomial random variable, an assumption which may not always be consistent with the observed variability.

Goovaerts (2005a) proposed as an alternative to estimate the semivariogram of the risk using the following population-weighted semivariogram of observed rates

(3) $${\hat{\gamma }}_{R}(h)=\frac{1}{2\sum_{i,j}^{N(h)}n\left(v_{i}\right)\times n\left(v_{j}\right)}\overset{N(h)}{\underset{i,j}{\sum }}n\left(v_{i}\right)\times n\left(v_{j}\right){\left[z\left(v_{i}\right)-z\left(v_{j}\right)\right]}^{2}.$$

The weighting scheme attenuates the impact of data pairs that involve at least one rate computed from small population sizes, revealing structures that might be blurred by the random variability of extreme values; compare Fig. 2E–F (green curve) to Fig. 2C–D. The weighting also tends to lower the sill of the semivariogram as well as the nugget variance.

### 3.1 Noise Filtering

Estimation and modeling of a semivariogram is typically a preliminary step to the application of what André would call “data expansion techniques”, such as kriging or simulation. At first, such techniques seem futile for cancer mortality maps, as there is no “unsampled location” *per se*. Keep in mind, however, that: (i) observed disease rates are unreliable because of the small number problem, and (ii) the magnitude of the noise varies spatially as a function of population size; see Sect. 2. Kriging with non-systematic measurement errors (Chiles and Delfiner 1999) thus turned out to be the perfect solution to filter this non-stationary noise.

The noise-filtered disease rate for a given area *v*_α_ , called risk, is estimated as a linear combination of the focal rate *z*(*v*_α_) and the rates observed in (*K*-1) neighboring entities *v*_*i*_

(4) $$\hat{r}\left(v_{\alpha }\right)=\lambda_{1}z\left(v_{\alpha }\right)+\overset{K}{\underset{i=2}{\sum }}\lambda_{i}z(v_{i}).$$

Weights λ_i_ are solutions of a traditional ordinary kriging system, except for an “error variance” term, a/*n*(*v*_*i*_), which is added only to the diagonal terms of the kriging matrix, resulting in smaller weights for less reliable data (i.e., rates measured over smaller populations *n*(*v*_*i*_))

(5) $$\overset{K}{\underset{j=1}{\sum }}\lambda_{j}\left[C_{R}\left(v_{i},v_{j}\right)+\delta_{ij}\frac{a}{n\left(v_{i}\right)}\right]+\mu \left(v_{\alpha }\right)=C_{R}\left(v_{i},v_{\alpha }\right)\;i=1,\ldots ,K\;\overset{K}{\underset{j=1}{\sum }}\lambda_{j}=1,$$

where *δ*_*ij*_=1 if i=j and 0 otherwise. The data-to-data and data-to-unknown covariance terms, *C*_*R*_(*v*_*i*_,*v*_*j*_) and *C*_*R*_(*v*_*i*_,*v*_*α*_), were at the time calculated as the value of the covariance of the risk for a lag equal to the Euclidean distance between the corresponding centroids; see Sect. 5 for advanced methods to account for spatial support. The term *a* equals $\bar{z}(1-\bar{z})-{\hat{\sigma }}_{R}^{2}$, leading to the binomial kriging estimator (Lajaunie 1991). In its first application, binomial kriging was used to produce a map of the risk of childhood cancer in the West Midlands of England (Oliver et al. 1998).

### 3.2 Application

**Figure 3.**
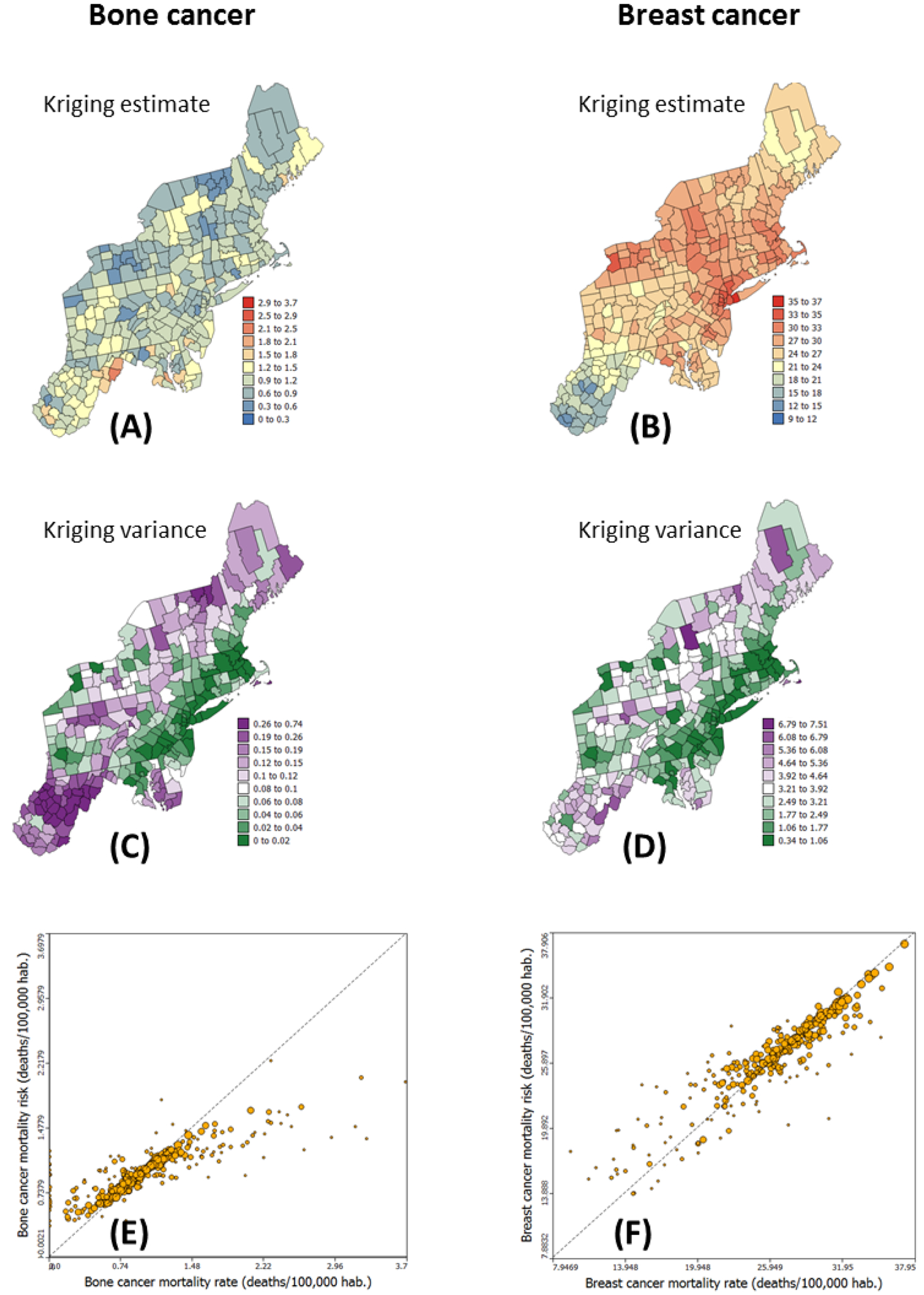
Results of smoothing of bone and breast cancer mortality maps of Fig. 2 using binomial kriging (A,B). The maps of kriging variance reflect the smaller uncertainty prevailing in highly populated counties along the Coast (C,D). These same counties show the smallest differences between kriging estimates and raw rates; see scatterplots where the size of each dot is proportional to the population living in that county (E,F)

Binomial kriging was used to filter the noise in the maps of bone and breast cancer mortality rates of Fig. 2. Kriging estimates and the associated kriging variance are mapped in Fig. 3A–D. The kriging variance, which is a function of population size, tends to be smaller along the Coast (e.g., New York, Boston, and Baltimore) where most of the population lives. On the other hand, the kriging variance is larger in less populated States, such as Maine (North) and West Virginia (South). The bottom scatterplots (Fig. 3E–F) illustrate the smoothing effect imparted by binomial kriging, in particular for sparsely populated counties depicted by smaller dots in the graph. The larger dots, which correspond to counties with greater population, lie closer to the 45-degree line as these mortality rates are more reliable and the impact of noise-filtering by binomial kriging is attenuated.

## 4. Poisson Kriging: from Fin Whales to Cancer Cases

The ad-hoc procedure of using population-weighted semivariograms with binomial kriging was presented during the Seventh International Geostatistics Congress that took place in Banff, Canada, in September 2004 (Goovaerts 2005a). My talk had been scheduled in a small session devoted to Environmental Applications, just following a paper presented by Pascal Monestiez on the spatial distribution of fin whales in Northwestern Mediterranean sea (Monestiez et al. 2005, 2006). I had known Pascal for over 25 years and always admired his rigorous statistical training and intellectual curiosity that pushed him to apply geostatistics to a wide variety of topics, such as fruit trees, river networks or spatial genetic structures. This scheduling turned out to be a perfect example of “*being at the right place at the right time*,” as Pascal’s talk was instrumental in solving the problems I had experienced with binomial kriging; see Sect. 3.

Pascal Monestiez was tackling the problem of characterizing the spatial distribution of rare wild species, in this case the fin whale, from unfrequent sightings and heterogeneous observation efforts (i.e., the total number of hours spent observing at a given location). To regularize such rare sightings the study area would typically be divided into small spatial cells for which counts and observation efforts would be summed. Their division would lead to an observation rate (i.e., fin whale sightings per hour) that would undergo the geostatistical analysis. The objective was to obtain reliable estimates of the frequency of sighting using neighboring observations and a new form of kriging, called Poisson kriging. As I was listening to Pascal, I immediately saw the analogy with the problem of analyzing cancer mortality rates; instead of counting whales, we were counting the number of deaths by cancer and the observation effort was simply the population size.

Poisson kriging deviates from binomial kriging in the definition of the error variance term in the kriging system (Eq. 5); *a* here equals $\bar{z}$, the population-weighted mean of the *m* rates. The other difference is the modeling of the semivariogram of the risk that relies on the following “Poisson” estimator

(6) $${\hat{\gamma }}_{R}(h)=\frac{1}{2\sum_{i,j}^{N(h)}\frac{n\left(v_{i}\right)n\left(v_{j}\right)}{n\left(v_{i}\right)+n\left(v_{j}\right)}}\overset{N(h)}{\underset{i,j}{\sum }}\left\{\frac{n\left(v_{i}\right)n\left(v_{j}\right)}{n\left(v_{i}\right)+n\left(v_{j}\right)}{\left[z\left(v_{i}\right)-z\left(v_{j}\right)\right]}^{2}-\bar{z}\right\},$$

where N(**h**) is the number of pairs of areas (*v*_*i*_,*v*_*j*_) whose population-weighted centroids are separated by the vector **h**. The squared spatial increments [*z*(*v*_*i*_)-*z*(*v*_*j*_)]^2^ are weighted by a function of their respective population sizes, n(*v*_*i*_)n(*v*_*j*_)/[n(*v*_*i*_)+n(*v*_*j*_)], a term which is inversely proportional to their standard deviations (Monestiez et al. 2006). More importance is thus given to the more reliable data pairs (i.e., smaller standard deviations). This semivariogram estimator turned out to cause fewer inconsistencies than Lajaunie’s estimator (Eq. 1), likely because cancer is a rare disease for which the Poisson distribution is a much more appropriate model than the binomial distribution. This estimator leads to semivariograms that bear similarity with the population-weighted estimator; compare blue and green curves in Fig. 2E–F.

## 5. Change of Spatial Support: Moving beyond Block Kriging

In the early applications of geostatistics to medical data (e.g., Cressie 1993; Oliver et al. 1993; Christakos and Lai 1997), aggregated rates were simply assigned to the geographic centroids of the administrative units, which enabled the implementation of a traditional “point” kriging approach (Eq. 5). This centroid-based implementation of kriging amounts to assuming that all the habitants in the unit live at the same location, and the measured rate thus refers to this specific location. This assumption is reasonable whenever the size and shape of geographical units, as well as the population distribution within those units, are uniform. The assumption of point measurement support becomes clearly inappropriate when the administrative units are counties or states, which calls for specific methods to incorporate the shape and size of those units in the analysis. This issue was also ignored by Monestiez et al. (2005) as the spatial support of their observations was small square cells of similar size.

Looking at the general formulation of kriging (Journel and Huibregts 1978, p. 306), it is clear that it can accommodate different spatial supports for both the data and the predicted unit. Using the terminology introduced by Gotway and Young (2002), geostatistics allows to tackle three different types of change of support: upscaling (e.g., point to area), downscaling (e.g., area to point), and side-scaling (e.g., two sets of overlapping areas, like ZIP codes to census tracts). Because of its mining origin the focus of geostatistics has mainly been to predict block averages from punctual data (upscaling by block kriging). Since then, the increase in computational power, coupled with the emergence of Geographical Information Systems (GIS), has made possible the analysis of multiple data sources recorded on supports of any shape and size. The terms “area-to-point” (ATP) and “area-to-area” (ATA) kriging were coined by Gotway and Young (2002) to denote the last two types of change of support. Goovaerts (2010) introduced the term “area-and-point” (AAP) kriging to combine areal and point data (e.g., geological units and soil measurements) for the prediction of point values.

Drawing from geostatistical concepts of incorporating into kriging data defined as linear combinations of point-support values, Kyriakidis (2004) showed in a quantitate geography context that: (i) area-to-point kriging yields coherent predictions, reproducing when aggregated the original area data no matter the point-level variogram model adopted, and (ii) several widely used methods for smoothing areal data, including Tobler’s celebrated pycnophylactic (density preserving) interpolation, could be regarded as particular cases of area-to-point kriging linked to implicit assumptions on point-level variogram models. To reduce the uncertainty associated with ATP kriging, secondary information at finer resolution can be incorporated in the disaggregation of areal data, e.g., using area-to-point residual kriging (Liu et al. 2008) or Poisson kriging with spatial drift (Bellier et al. 2009). Similarly, Gotway and Young (2007) used kriging to map the number of low birth weight (LBW) babies at the Census tract level, accounting for county-level LBW data and covariates, such as a fine grid of ground-level particulate matter concentrations and tract population.

### 5.1 ATA and ATP Poisson Kriging

Thanks to the development of Poisson kriging and separate advances in the field of change of spatial support, it seemed I had all the pieces to incorporate the size and shape of administrative units, as well as the population density, into the filtering of noisy mortality rates and the mapping of the corresponding risk at a fine scale (i.e., disaggregation). The techniques were appropriately called area-to-area and area-to-point Poisson kriging (Goovaerts 2006a). Point-to-point covariances in the kriging system (Eq. 5) are simply replaced by area-to-point or area-to-area covariance terms, depending on the type of change of spatial support. Those covariances are numerically approximated by averaging the point-support covariance *C*_*R*_(**h**) computed between any two locations discretizing the two geographical units; e.g. for areas *v*_*i*_ and *v*_*j*_

(7) $${\bar{C}}_{R}\left(v_{i},v_{j}\right)=\frac{1}{\sum_{l=1}^{P_{i}}\sum_{l^{\prime }=1}^{P_{j}}w_{ll^{\prime }}}\overset{P_{i}}{\underset{l=1}{\sum }}\overset{P_{j}}{\underset{l^{\prime }=1}{\sum }}w_{ll}C_{R}\left(s_{l}-s_{l^{\prime }}\right),$$

where *P*_*i*_ and *P*_*j*_ are the number of points used to discretize the two areas *v*_*i*_ and *v*_*j*_, respectively. The weights *w*_*ll’*_ allow accounting for uneven population density within large geographical units. They are computed as the product of population sizes assigned to each discretizing point **s**_*l*_ and **s**_*l’*_ (e.g., assuming a uniform population density within a census tract or smaller census geographies)

$$w_{ll^{\prime }}=n\left(s_{l}\right)\times n\left(s_{l^{\prime }}\right)\;\;\text{with}\;\;\overset{P_{i}}{\underset{l=1}{\sum }}n\left(s_{l}\right)=n\left(v_{i}\right)\text{ and }\overset{P_{j}}{\underset{l^{\prime }=1}{\sum }}n\left(s_{l^{\prime }}\right)=n\left(v_{j}\right).$$

The practical implementation of ATA and ATP kriging required, however, knowledge of the point-support covariance of the risk *C*_*R*_(**h**), or equivalently the semivariogram *γ*_*R*_(**h**), which cannot be inferred directly since only areal data are available. Derivation of a point-support semivariogram from the experimental semivariogram of areal data is called “deconvolution”, an operation that is frequent in mining and was already described in much detail in Journel and Huibregts (1978). However, in typical mining applications all blocks have the same size and shape, which makes the deconvolution reasonably simple using analytical procedures. In addition, the aggregation of rate data within each geographical unit is spatially weighted as it depends on the spatial distribution of population that can be extremely heterogeneous. This time I was on my own and had to come up with a solution. Using the brute force of the computers (another of André’s favorite expressions), I designed an iterative procedure whereby one seeks the point-support model that, once regularized, is the closest to the model fitted to areal data (Goovaerts 2008). This empirical procedure actually automated the heuristic approach proposed by Journel and Huijbregts (1978), whereby the user was advised to manually modify the parameters of the point support model until its regularization is close to the model fitted to areal data. Yet, deconvolution is an inverse problem, and as such, there are multiple point-support models that once regularized will yield the model fitted to areal data. Deconvolution is still an active area of research; in particular the development of a Bayesian approach to ATP kriging in order to introduce prior knowledge about the semivariogram model parameters, as well as accounting for uncertainty about these parameters in the prediction (Truong et al. 2014; Brus et al. 2018).

### 5.2 Application

**Figure 4.**
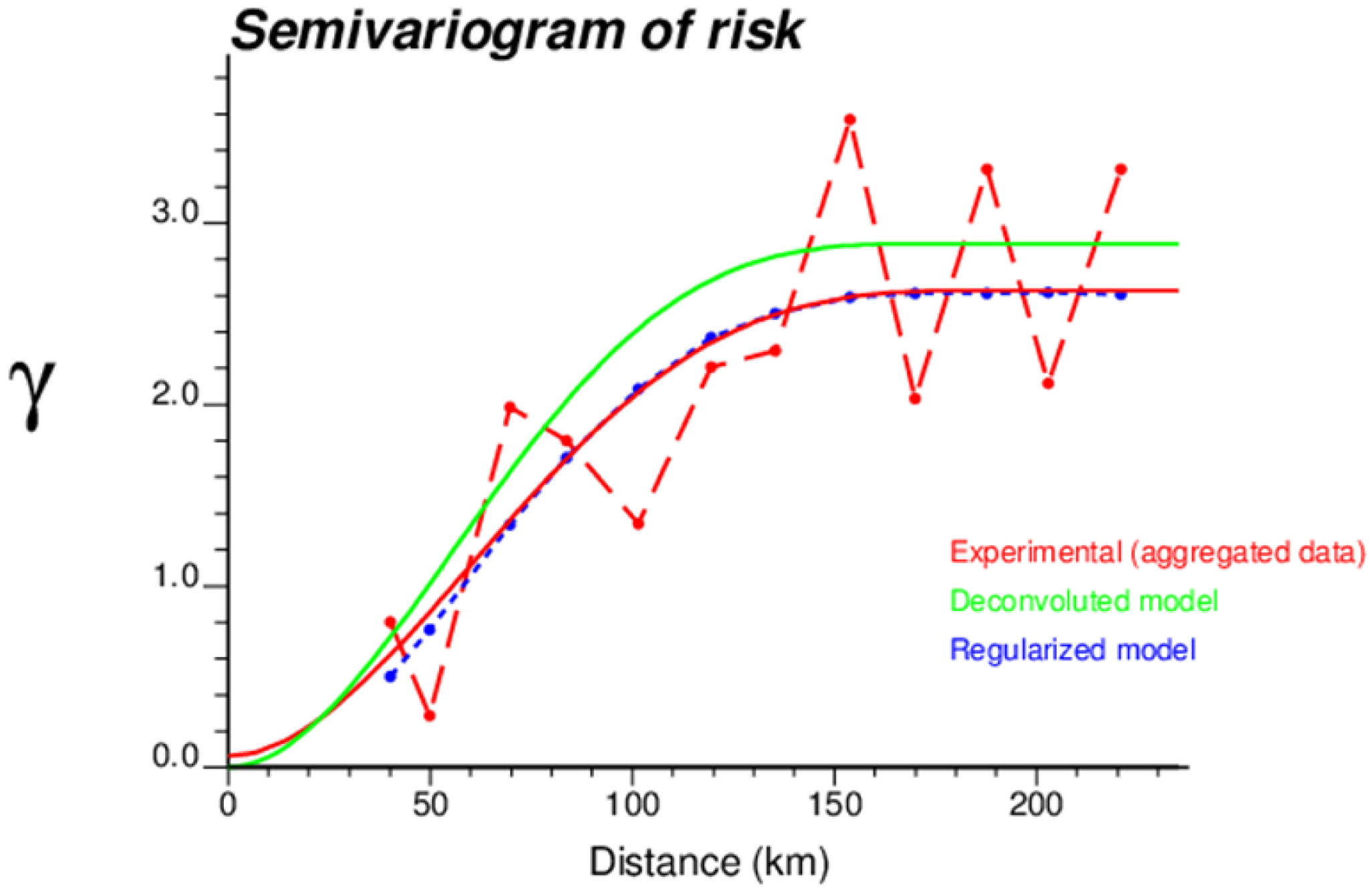
Experimental semivariogram of the risk estimated from county-level rate data, and the results of its deconvolution (top green curve). The regularization of the point support model yields a curve (blue dashed line) that is very close to the experimental one (red curve)

**Figure 5.**
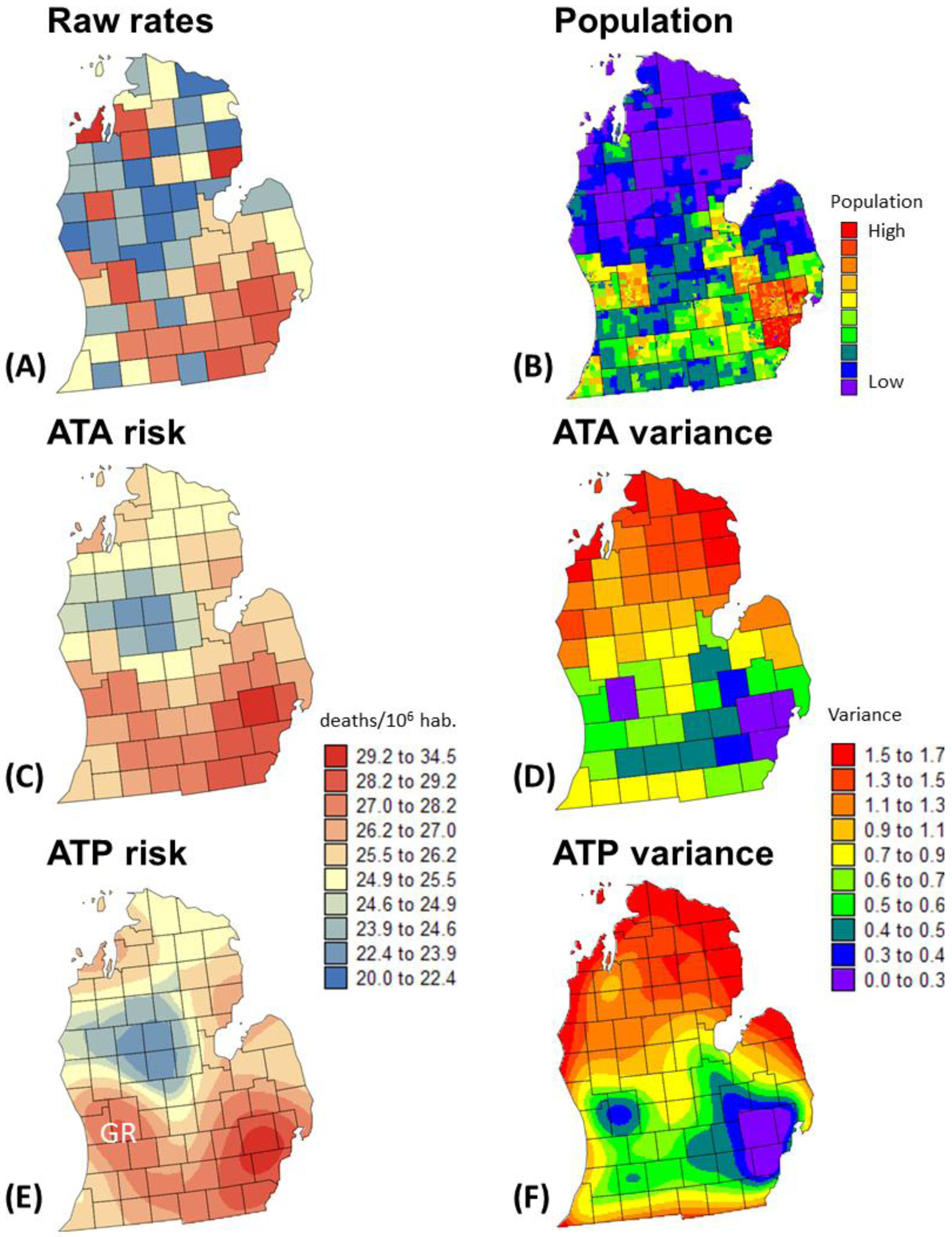
Illustration of the concept of change of spatial support for health outcomes. Maps of white female breast cancer mortality risk (units = number of deaths/100,000 habitants) and associated prediction variance estimated at the county level (ATA kriging) or at the nodes of a 2 km spacing grid (ATP kriging). The information available consists of county-level mortality rates (A) and census tract population data (B). Maps A,C & E share the same color scale whereas the same color legend is used for the two maps of kriging variance

Figures 4 and 5 illustrate the application of geostatistics to breast cancer mortality rates recorded over the 1970–1994 period for each county of Michigan Lower Peninsula. Figure 4 shows the experimental and model semivariograms of risk computed from areal rate data using Eq. 6. This model is then deconvoluted and, as expected, the resulting model (green curve) has a higher sill since the punctual process has a larger variance than its aggregated form. Its regularization (short blue dashed line) yields a semivariogram model that is close to the one fitted to experimental values (red curve), which validates the consistency of the deconvolution.

The deconvoluted model was used to estimate mortality risk at the county level (ATA kriging) and to map the spatial distribution of risk within counties (ATP kriging). Both maps are much smoother than the map of raw rates (Fig. 5A) since the noise due to small population sizes is filtered. In particular, the highest rate recorded in the sparsely populated Leelanau peninsula (NW) dropped from 34.5 to 26.3 deaths/100,000 habitants. ATA kriging highlights large cancer mortality rates in the Detroit area (SE). ATP kriging (Fig. 5E) allows a finer analysis, suggesting the existence of another pocket of high risk near Grand Rapids (GR in map), the economic and manufacturing center of West Michigan. This feature, which was blurred by the county-level aggregation, is important for designing prevention strategies after confirmation through local field data. By construction, aggregating the ATP kriging estimates within each county using the population density map yields the ATA kriging map. The map of ATA kriging variance essentially reflects the higher confidence in the mortality risk estimated for counties with large populations in SE Michigan. The population distribution can, however, be highly heterogeneous in counties with contrasted urban and rural areas (Fig. 5B). This information is incorporated in ATP kriging. As expected, the ATP kriging variance is larger than the ATA kriging variance (Fig. 5F).

## 6. Indicator Kriging and the Spatial Analysis of Individual-level Data

Thanks to André’s constant support and encouragement, my two-year stint at Stanford was a very productive time, with the preparation of six papers and a book. André Journel agreed to co-author only two of these papers, stressing the importance for prospective faculty members to have single author publications under their belt. This unselfish attitude sometimes made me question whether my research was good enough, and I was delighted the first time André asked me to write his name next to mine on top of a manuscript (Goovaerts and Journel 1995). This paper was describing two indicator algorithms to incorporate soil map information into the prediction of continuous soil properties. Upon André’s recommendation, the manuscript had been peer-reviewed by four senior geostatisticians before its submission to the European Journal of Soil Science, which at the time was the leading geostatistical journal in the field of soil sciences. Such a thorough preparation, coupled with the prestige of my co-author, made me believe that the review process would be a walk in the park. Alas, this paper took two years to be published, being rejected twice by the same reviewer who confided to the Editor-in-Chief that “*the analysis had no firm theoretical basis, and it would be a pity and even destructive if such a fuzzy paper appeared on such an important topic.*” Since then, indicator kriging has always occupied a very special place in my geostatistical toolbox.

Whenever possible, it is beneficial to avoid the tedious, arbitrary and inherently information-wasteful aggregation of health outcomes within geographical units and to process directly the point-based data (Bithell 2000). In addition to the greater accuracy in the location of health outcomes, the analysis of individual-level data can often capitalize on detailed information on residential history and a large number of potential risk factors. Mapping this type of data presents, however, challenges besides legal and technical issues related to the privacy of personal health information as well as address-matching errors that can lead to spatial misalignment. A simple location map of health events or cases is misleading unless the population density is spatially uniform. It is however unrealistic to expect the coordinates of every member of the population at risk to be known. One exception is the analysis of health outcomes (e.g., low birth weight, cancer tumor stage at diagnosis) for which the population at risk can be enumerated and geocoded fairly easily (e.g., newborns or patients diagnosed with cancer).

### 6.1 Indicator Semivariogram

The information about each case, referenced geographically by its residence’s spatial coordinates **u**_*i*_=(x_*i*_,y_*i*_), takes the form of an indicator of occurrence of an event; for example for the case of early/late stage diagnosis of cancer

(8) $$\text{ind}\left(u_{i}\right)=\;\{{}_{0\;\text{otherwise}.}^{1\;\text{ if late stage diagnosis}}$$

Spatial patterns can be characterized using the indicator variogram $2{\hat{\gamma }}_{I}(h)$ which measures how often the stage of diagnosis of two cases a vector **h** apart is different. In presence of spatial clusters of early or late-stage diagnosis, the semivariogram is expected to increase with the lag **h** and reaches a plateau at a distance, called range, which corresponds to the average size of these clusters. If these clusters are non-circular, different ranges will be observed along different directions, a situation referred to as spatial anisotropy. The study of indicator semivariograms can thus provide important information about the nature and scale of the process responsible for the spatial distribution of cancer stages at diagnosis.

### 6.2 Spatial Contouring of Risk

Estimation and mapping of the spatial risk function requires the computation of the ratio of the case density to the population density. The most straightforward approach is to use „kernel density estimation methods’, whereby the number of cases and the total number of individuals at risk are summed within sliding windows and their ratio defines the rate assigned to the center (i.e., grid node) of that window (James et al. 2004). The operation is repeated for each grid node, leading to the creation of isopleth maps of, for example, late-stage cancer rates (ratio of number of late-stage cancer cases to total number of people diagnosed with that cancer).

The estimator of the spatial risk function *λ* at any location **u** can be expressed as a linear combination of *n*(**u**) indicator-coded observations falling within a window W(**u**)

(9) $$\hat{\lambda }(u)=\overset{n(u)}{\underset{i=1}{\sum }}\omega_{i}ind\left(u_{i}\right)\;\;\text{with}\;\;\overset{n(u)}{\underset{i=1}{\sum }}\omega_{i}=1.$$

Many variants of the same principle have been proposed since its initial application in epidemiology. For example, the size of the window or spatial filter can remain constant over the study area (e.g., Rushton et al. 2004) or the spatial filter size is locally adjusted (spatially adaptive filters) to ensure the capture of a constant population size (e.g., Talbot et al. 2000). Main differences between estimators reside in the choice of weights *ω*_*i*_ ; for example, all the observations within the spatial filter are weighted the same, i.e., ω_*i*_=1/n(**u**), (Talbot et al. 2000) or they are weighted according to their proximity to the center of the filter (Rushton 2003).

Whereas the computation of empirical estimates of type (Eq. 9) is very straightforward, critical information regarding the pattern of spatial dependence of observations, the data geometry (i.e., isolated cases in rural areas versus clustered cases in urban settings) and the spatial support of the observation (individual point data versus cases aggregated into small census areas) is ignored in the process. So, indicator kriging was a no-brainer to compute the weights *ω*_*i*_ assigned to each indicator (Goovaerts 2009a).

### 6.3 Application

**Figure 6.**
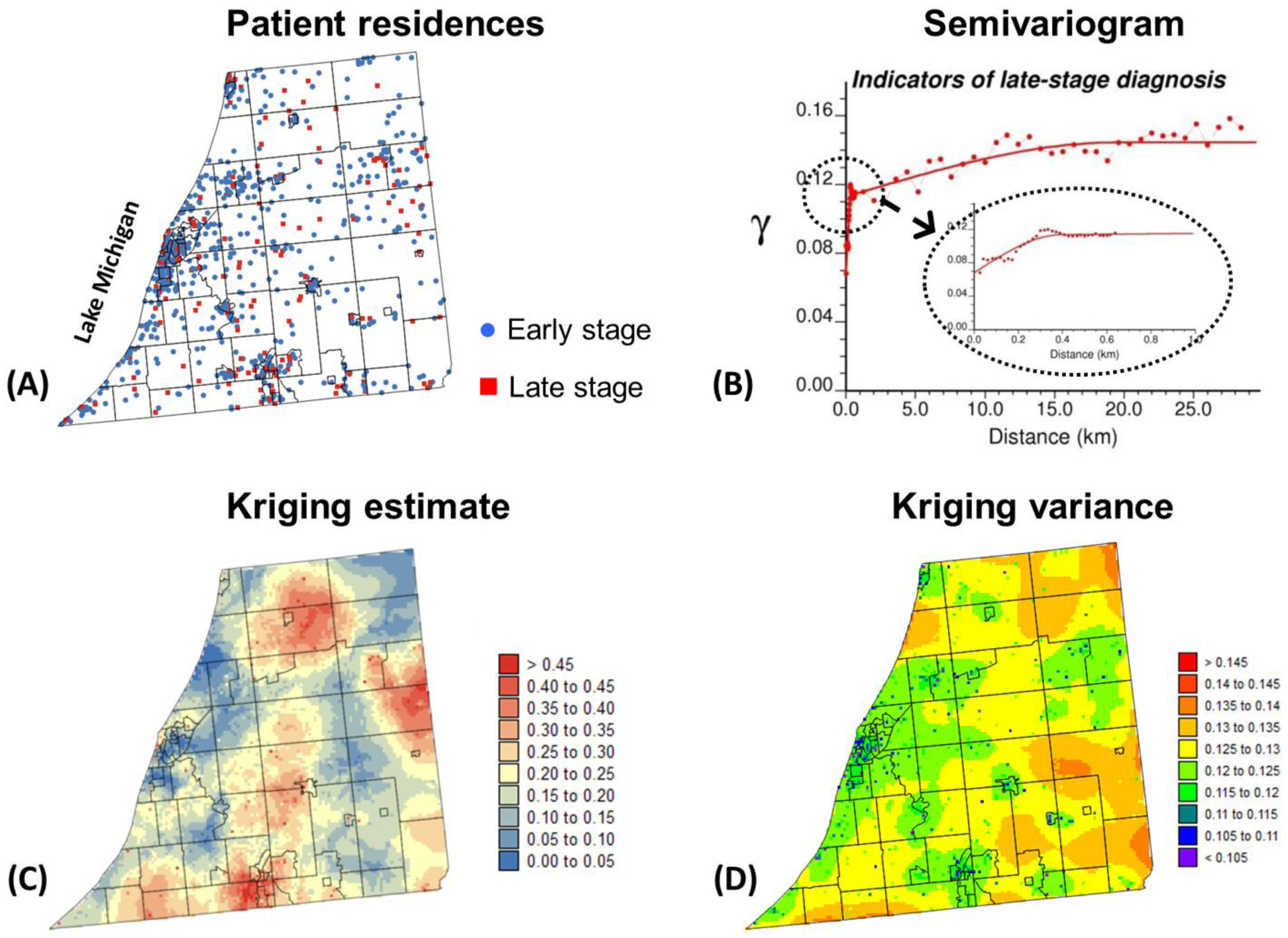
Information available for mapping the probability of late-stage breast cancer diagnosis in three Michigan counties: (A) Location of 937 patient residences with indicators of stage at diagnosis, (B) Semivariogram of indicators of late-stage diagnosis, (C) Probability map created using ordinary indicator kriging, (D) Map of the prediction variance associated with the indicator kriging estimate

Figure 6 illustrates the application of indicator geostatistics to the mapping of probability of late-stage diagnosis of breast cancer within three Michigan counties. The population includes 64–75 year-old white women diagnosed with breast cancer over the period 1985–2002. The first step was to code each cancer case (*m*=937) as 1 for late-stage diagnosis and 0 otherwise (Fig. 6A, exact locations shuffled for confidentiality reasons). To account for the wide range of separation distances between cancer cases (from a few meters to 112 km), the indicator semivariogram in Fig. 6B was computed using two series of lag classes: 37 lags of 30 meters to characterize the small-scale variation of the data and 37 lags of 800 meters to look at the regional pattern. The spatial variability is clearly nested: most of the variance occurs over short distances (1st range = 397 meters) and is superimposed on a regional structure with a range of 19.57 km. Late detection cases do not occur randomly in space; however, individual-level factors, such as age or family history, generate a large variability over very short distances.

Based on the spatial autocorrelation, the late-stage cancer risk and the variance of prediction errors were estimated at each node of a 600 m spacing grid, using indicator kriging (IK) and 32 closest cases. The corresponding maps (Fig. 6C–D) highlight the substantial change in proportion of late-stage diagnosis (from 0 to 0.56) across the three counties, with more uncertainty (larger kriging variance) in rural areas where fewer cases were diagnosed.

## 7. How does Stochastic Simulation Fit into the Picture?

Because of my formal training in a department of biometry, I was accustomed to performing statistical tests and interpreting significance levels before heading to Stanford. On the other hand, André Journel was never a big proponent of hypothesis testing. In particular, he insisted that stationarity was not a hypothesis that could be proven (or refuted) by statistical tests (Journel 1989). Instead, it was a necessarily subjective decision that the modeler had to make when data were too sparse to do otherwise. André elaborates further on the illusive quest for objectivity in his plenary talk at the 1996 Geostat conference in Wollongong (Journel 1997).

I agree wholeheartedly with him that most statistical tests aimed at providing “objectivity” in data analysis and decision-making rely on models and assumptions, such as the I.I.D. assumption (data are independent, identically distributed) or the multivariate Gaussian model, that are inconsistent with most datasets. I am always perplexed by a geostatistical analysis that starts with a test of normality of the population distribution as this test is based on the assumption of independence of observations, which would make any subsequent geostatistical treatment pointless. Nevertheless, epidemiologists frequently conduct hypothesis testing to detect local areas with significantly higher mortality or incidence rates (cluster detection), to identify borders where these rates vary abruptly (boundary detection), and to find covariates that impact significantly the health outcome (regression analysis).

### 7.1 Local Cluster Analysis (LCA)

Cluster detection is often based on the following LISA (Local Indicator of Spatial Autocorrelation) statistic (Anselin 1995)

(10) $$\text{LISA}\left(v_{\alpha }\right)=\left[\frac{z\left(v_{\alpha }\right)-m}{s}\right]\times \left(\overset{J\left(v_{a}\right)}{\underset{j=1}{\sum }}\frac{1}{J\left(v_{\alpha }\right)}\times \left[\frac{z\left(v_{j}\right)-m}{s}\right]\right),$$

which quantifies the similarity between the rate *z* measured within area *v*_α_ and those recorded in J(*v*_α_) adjacent areas *z*(*v*_j_) (e.g., units sharing a common border or vertex with the focal area *v*_α_). All values are standardized using the mean *m* and standard deviation *s* of the dataset. A positive LISA thus indicates the presence of a spatial cluster (positive autocorrelation): the focal and neighborhood averaged rates jointly exceed the global mean *m* (High-High, HH cluster) or are jointly below *m* (Low-Low, LL cluster). On the other hand, a negative LISA indicates a negative local autocorrelation and the presence of a spatial outlier where the focal value is much lower (Low-High, LH outlier) or higher (High-Low, HL outlier) than the average of surrounding values.

The magnitude of the LISA statistic informs on the extent to which focal and neighborhood values differ. To test whether this difference is significant or not, a Monte Carlo simulation is conducted, which traditionally consists of sampling randomly and without replacement the sample histogram and computing the corresponding simulated neighborhood averages. This operation is repeated many times (e.g., *M*=999 draws) and these simulated values are multiplied by the focal value to produce a set of *M* simulated values of the LISA statistic for the unit *v*_α_. This set represents a numerical approximation of the probability distribution of the LISA statistic at *v*_α_, under the assumption of “spatial independence”. The observed statistic (Eq. 10) is compared to the probability distribution, enabling the computation of the *p*-value of the test. Every geographical unit where the *p*-value is lower than the significance level, after correcting for multiple testing, is classified as a significant spatial cluster or outlier.

**Figure 7.**
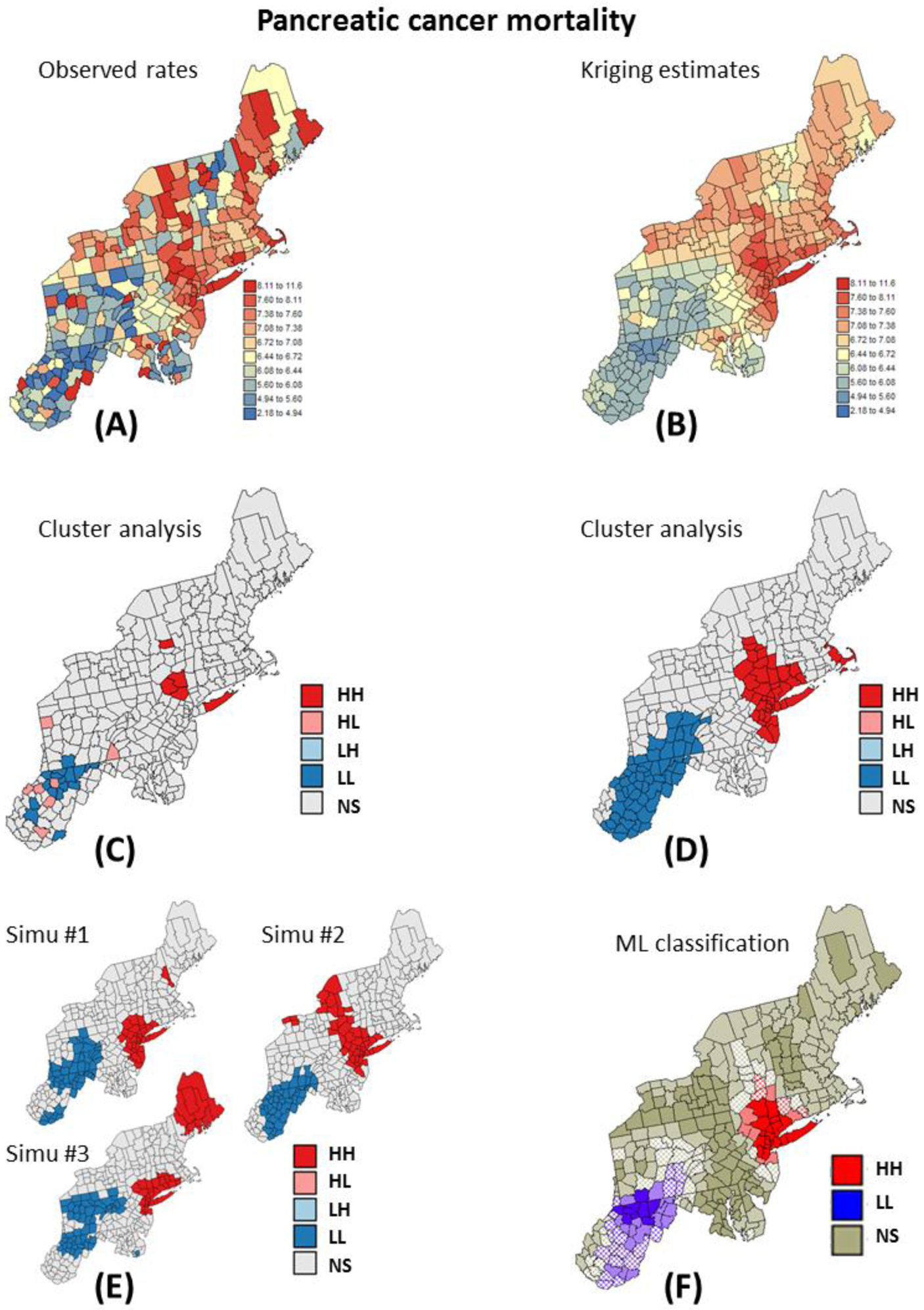
Maps of pancreatic cancer mortality rates (units = number of deaths per 100,000 habitants) recorded during the 1950–1969 period for 295 counties of 12 New England States before (A) and after (B) noise-filtering by Poisson kriging. Results of LCA conducted on: original map (C), kriged map (D), and three simulated maps (E). Most likely (ML) classification inferred from 500 simulated maps (F); see legend description in text

An example of LCA is provided for pancreatic cancer mortality rates recorded in 295 counties of 12 New England States (1950–1969 period). Because pancreatic cancer is less prevalent than other cancers, such as lung or breast cancer, mortality rates are prone to the small number problem and exhibit strong spatial variability (Fig. 7A). Besides a few clusters of low and high values, located respectively in West Virginia and New York, several counties are classified as high-low outliers (Fig. 7C), indicating that their mortality rates are significantly higher than the mortality recorded in adjacent counties. These results must, however, be interpreted with caution given the small population sizes of some of these counties. Indeed, the HL tests become non-significant when the analysis is conducted on the map of kriged risks (see Fig. 7D).

### 7.2 Propagation of Uncertainty

The application of the LISA statistic, albeit widespread in epidemiology, suffers from two limitations. First, the uncertainty attached to rates (i.e., small number problem) is ignored; some studies proposed to apply the LISA statistic to noise-filtered rates, yet it tends to inflate artificially the size of the clusters since the smoothing imparts autocorrelation to risk estimates. For the example of pancreatic cancer, the LL and HH clusters expanded into much larger clusters on the classified risk map, while all seven spatial outliers were smoothed out (Fig. 7D). Second, the test is based on the null hypothesis of spatial independence (SI) of observed rates and, provided the population sizes of areal units are fairly homogeneous, the assumption of constant or spatially uniform risk; since some spatial pattern is almost always present, rejecting this hypothesis has little scientific value. It didn’t take me long to realize the potential of stochastic simulation for tackling both challenges (Goovaerts 2006b).

The first issue bears similarity with the problem of propagating spatial uncertainty through a non-linear transfer function, a problem that the geostatistical community solved a long time ago. A solution consists of conducting a LCA on an ensemble of *K* simulated maps of rates, leading to a set of *K* classified maps. Under the assumption that all realizations are equally-probable, the probability of occurrence of the two main types of clusters (LL, HH) can then be computed from sample frequencies for each geographical unit.

Five hundred realizations of the spatial distribution of risk values were simulated using the approach described in Goovaerts (2006b) before undergoing a LCA. Figure 7E shows the results for three realizations. From one realization to another, the shape and position of local clusters can change substantially. For example, the southern part of the cluster of low risk values detected on the kriged map (Fig. 7D) becomes non-significant on some realizations. Differences among realizations depict the uncertainty attached to the classification. For example, in Maine and the Northwestern part of the State of New York, the simulated risk for pancreatic cancer takes a wide range of values, emphasizing the lack of reliability of the clusters of high values detected in these sparsely populated regions on some realizations. On the other hand, a cluster of high values is consistently found for the New York area across all simulated classifications.

The information provided by the set of *K* LCAs is summarized in Fig. 7F. The color code indicates the most frequent classification (maximum likelihood = ML) of each county across the 500 simulated maps. The shading reflects the probability of occurrence or likelihood of the mapped class. Solid shading corresponds to classifications with high frequencies of occurrence (i.e., likelihood > 0.9), while hatched counties denote the least reliable results (i.e., likelihood < 0.75). This coding is somewhat subjective but leads to a clear visualization of the lower reliability of the cluster of low mortality in Western Virginia and Southern Pennsylvania relatively to the cluster of high risk identified in the heavily populated New York area. As intuitively expected, less reliable results are found for counties located on the edge of the clusters.

### 7.3 Neutral Model

The concept of “neutral model” (Goovaerts and Jacquez 2004) allows the testing of more interesting hypotheses by replacing the null hypothesis of spatial randomness and uniform risk by models that account for spatial patterns and a priori information on the underlying risk. The problem then is to identify spatial patterns above and beyond that incorporated into the neutral model, enabling, for example, the identification of “hot spots” beyond background variation in a pollutant or the detection of clusters beyond regional variation in the risk of developing cancer. Goovaerts and Jacquez (2004) proposed to use sequential Gaussian simulation to generate realizations of the spatial distribution of mortality rates under increasingly stringent conditions: (i) reproduction of the sample histogram, (ii) reproduction of the pattern of spatial autocorrelation modeled from the data, (iii) incorporation of regional background obtained by kriging of the local mean, and (iv) integration of local trends in cancer rates inferred from the calibration of an exposure model.

**Figure 8.**
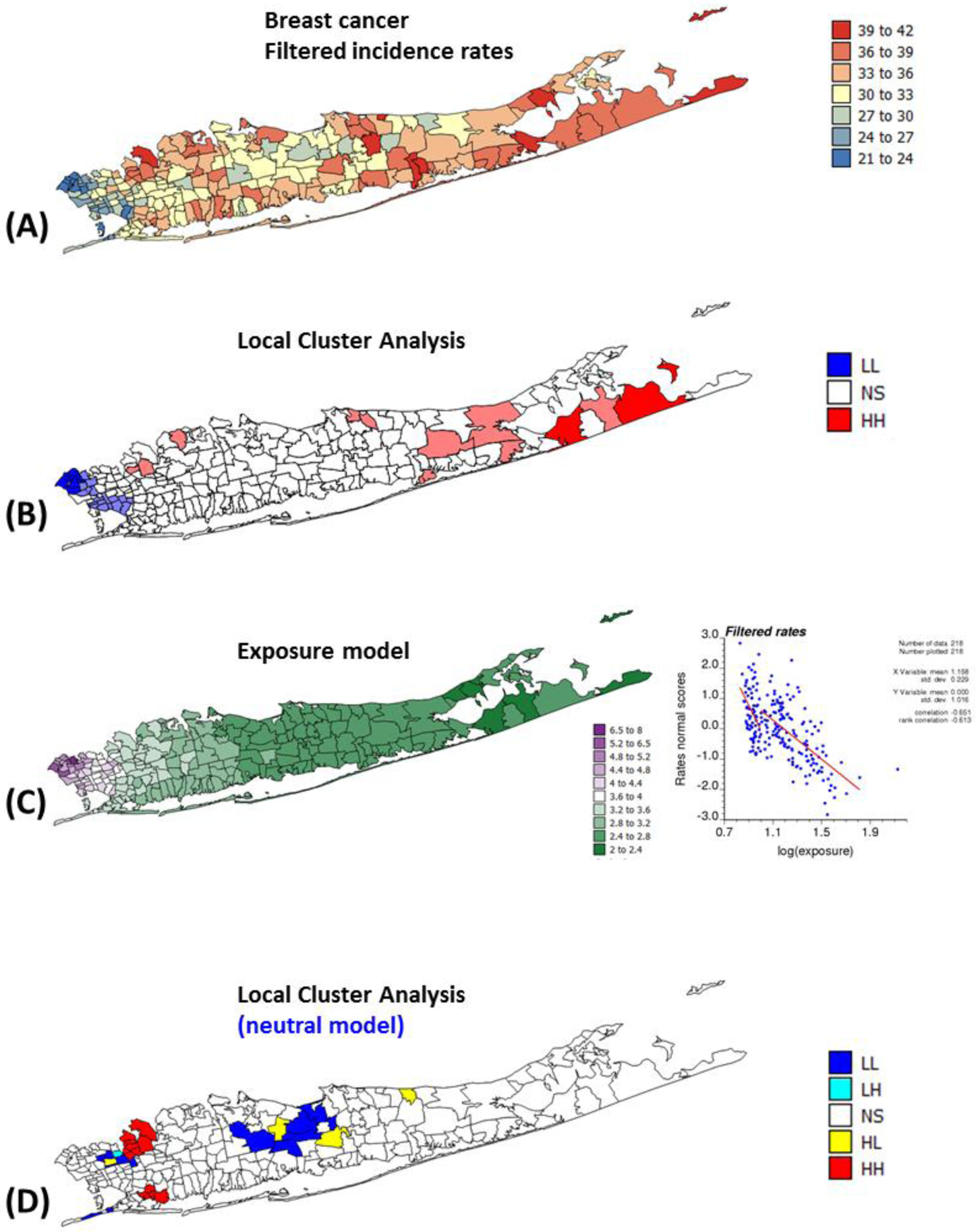
(A) Map of filtered incidence rates of breast cancer (units = cases/100,000 habitants) recorded during the period 1993–1997 in 214 ZIP codes on Long Island, New York. (B) LCA results conducted under the hypothesis of spatial independence; solid shading denotes fewer significant tests when accounting for spatial dependence. (C) Background risk inferred from the environmental exposure model, and LCA results to detect spatial pattern above and beyond this risk (D)

Figure 8A shows the incidence rate of breast cancer recorded for each ZIP code on Long Island during the period 1993–1997. The rates, which were smoothed using binomial kriging, range from 21 to 42 cases per 100,000 women and increase in the eastern part of the island. This is confirmed by the local cluster analysis (Fig. 8B) which indicates significant clusters of high values in the central and eastern parts of the island, while low values are confined to the western part. Solid shading denotes the subset of ZIP codes that tested significant when using spatially correlated neutral models. This more realistic neutral model leads to larger *p*-values and a substantial reduction in the number of geographical units declared significant clusters. This result confirms an earlier finding that the SI hypothesis often leads to an over-identification of the number of significant spatial clusters.

The integration of local trends in the generation of neutral models was achieved using simple kriging with spatially varying local means instead of a global constant mean to derive the mean and variance of local probability distribution functions (see Goovaerts 2005b for more details). These local means were derived by calibration of an airborne carcinogen exposure model described in Jacquez and Greiling (2004). In this latter case, the relationship between exposure and incidence rates was modeled using linear functions fitted separately to low exposures observed in the eastern part of Long Island and high exposures in the western part (Fig. 8C). When the constraint of local conditioning of neutral models is superimposed to the reproduction of spatial autocorrelation, the approach allows one to detect local departures from the incidence background specified by the user, leading to a very different map of spatial clusters and outliers. In particular, Fig. 8D reveals a series of ZIP codes that are significant high clusters in the northwestern part of the island. Cancer incidences in these ZIP codes are higher than expected under the environmental exposure model and should warrant further investigation to identify additional cofactors.

## 8. As the Crow Flies or as the Cancer Patient Travels?

One of the key concepts in spatial statistics is captured by Tobler’s first law of geography that states “*Everything is related to everything else, but near things are more related than distant things.*” Practical implementation of this concept requires a mathematical description of “near” and “distant”, as well as “related”. The most common and simple measure of spatial proximity is the Euclidean or “as the crow flies” distance between two objects. However, as stressed by Oyana and Margai (2016), spatial proximity is a function of both the distance and the degree of connectivity between objects, people or places in the geospatial database. Measures can then be based not only on linear distance but also to account for physical boundaries, costs, time and network within the system.

In the presence of barriers (e.g., mountains, water bodies, buildings), the distance between two locations could be identified with the minimum distance that has to be traveled without crossing any barriers, also known “as the fish swims” distance. In the situation when barriers or areas are semi-permeable so that some regions are harder (or easier) to cross, this heterogeneity can be modeled using a cost surface (e.g., units of cost, risk, or travel time), representing how difficult it is to cross a given portion of an area. Spatial proximity can then be measured using cost-weighted distances (Krivoruchko and Gribov 2004) and two locations are connected by the least-cost path (López-Quílez and Muñoz 2009) or the shortest-path distance (SPD).

### 8.1 Space Transformation

Several authors (Curriero 2006; Ver Hoef 2018) showed that most semivariogram models commonly applied in geostatistics are not valid for distance measures other than the Euclidean distance. One approach to ensuring the validity of a semivariogram model for non-Euclidean distances is to transform the coordinates and conduct the analysis in a new Euclidean space. This operation, known as “isometric embedding” in a Euclidean space, is a distance-preserving transformation whereby the distance between objects (e.g., sampled locations, grid nodes) in the new Euclidean space is equal to the distance between objects in the original space. A general approach that applies to complex geometries (e.g., dissected coastline, road network) and any type of metric (e.g., travel time, least-cost path) is to conduct a multidimensional scaling (MDS) of a matrix of distances or dissimilarities (Løland and Høst 2003; Boisvert et al. 2009). The objective is to project the objects in a low-dimensional space such that the between-object distances are preserved as well as possible; note that the original coordinates of the objects are not required, only their separation distances in the original space.

**Figure 9.**
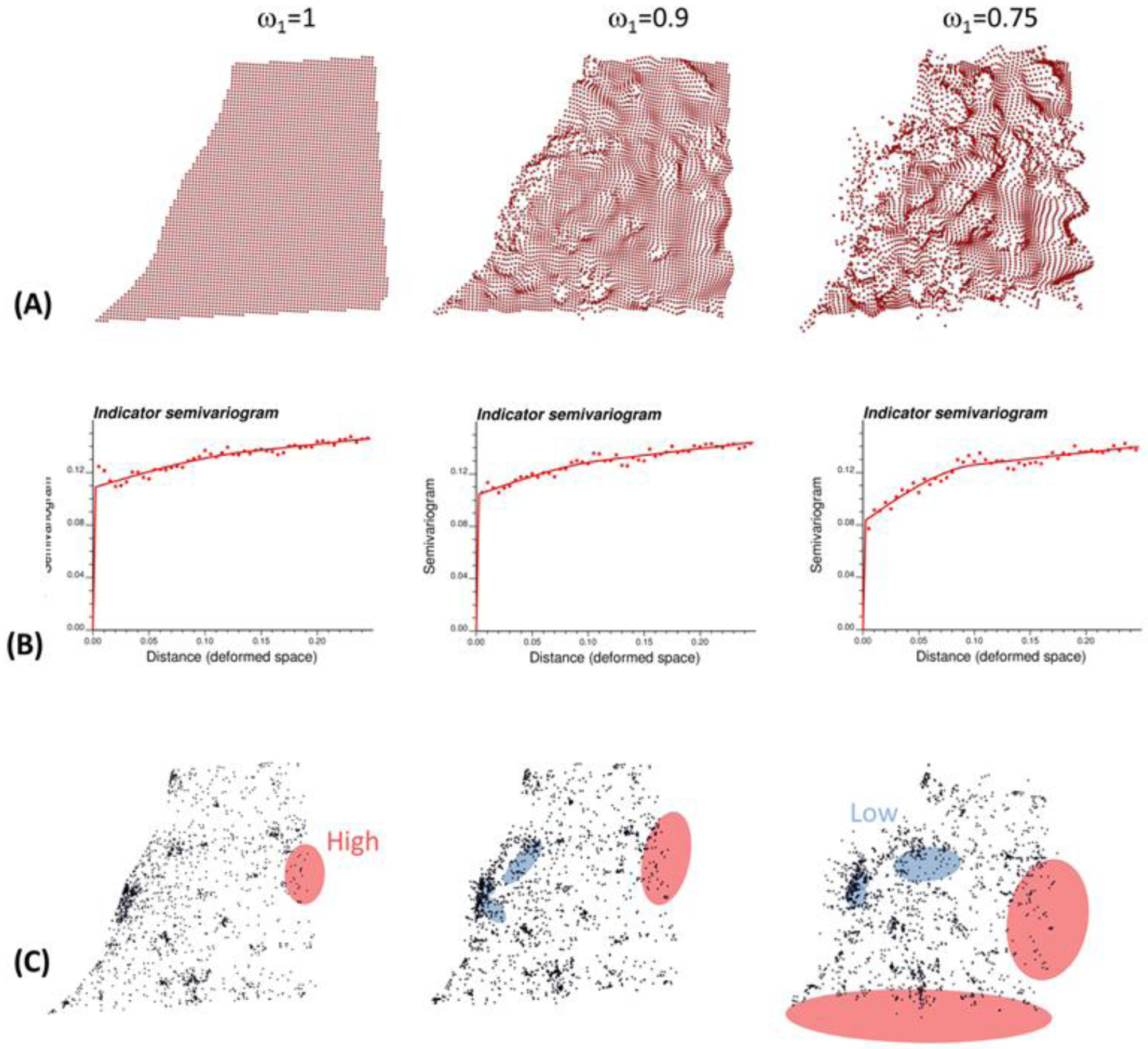
Illustration of the spatial deformation approach using 2,118 breast cancer cases recorded in three counties of Michigan: (A) deformation of the spatial interpolation grid obtained for different values of the weight *w*_1_ assigned to the reproduction of Euclidean distances, and the corresponding: (B) indicator semivariogram of late-stage diagnosis, and (C) clusters of high and low frequency of late-stage diagnosis detected using a spatial scan statistic

A limitation of this transformation is that it ignores the structures of spatial dependence and focuses only on the reproduction of spatial proximities. Local structures of spatial dependence (i.e., non-stationary correlation function) can be accounted for by applying MDS to a matrix of sample spatial dispersion, resulting in a spatial deformation where the domain is stretched in regions of relatively lower spatial correlation (i.e., higher dispersion), while contracting it in regions of higher spatial correlation; see Fig. 9A for an example. This idea was introduced by Sampson and Guttorp (1992) who computed the spatial dispersion between each pair of sampled locations as the Euclidean distance between time series. This approach requires the availability of space-time data (i.e., temporal replication) and assumes that the spatial dispersion is constant across time. Fouedjio et al. (2015) relaxed the strong assumption of replication by using a kernel weighted local average of squared increments between any two pair of sampled locations, thereby replacing observations recorded at different times (temporal replication) by observations recorded in close proximity (replication across space). The same authors proposed to combine spatial proximities with spatial dispersion (i.e., weighted average of the two metrics) to reduce the risk that the deformation function folds.

Recently, I proposed to combine three types of distance metrics to quantify dissimilarities *D*_*ij*_ between any two observations i and *j*

$$D_{ij}=w_{1}\times ED_{ij}+w_{2}\times G_{ij}+w_{3}\times FB_{ij}\;\;\text{with}\;\;w_{1}+w_{2}+w_{3}=1,$$

where *ED*_*ij*_ is the Euclidean distance, *G*_*ij*_ is the spatial dispersion estimated using a kernel weighted average of squared increments between any two observations within a given distance of *i* and j, and *FB*_*ij*_ is the distance in the feature space (e.g., Mahalanobis distance between census-tract poverty levels and access to mammography clinics quantified by the floating catchment method (Luo 2004) for the example of Fig. 9).

### 8.2 Application

The spatial deformation approach is illustrated, using 2,118 individual-level data on breast cancer stage of diagnosis recorded in three counties of Michigan (Fig. 9). The first row illustrates how the spatial interpolation grid is deformed as the weight *w*_1_ assigned to the reproduction of Euclidean distances decreases while the weight assigned to spatial dispersion and distance in feature space increases, stretching regions of relatively lower spatial correlation while contracting regions of higher correlation. Accounting for spatial dispersion improves the shape of semivariogram (Fig. 9B) in the deformed space and leads to the detection of different clusters of high and low frequency of late-stage diagnosis (Fig. 8C), using the spatial scan statistic (Kulldorff 2018). This statistic aims to identify a significant excess (or deficiency) of cases of late-stage diagnosis within a moving elliptical window that visits all residential locations, increasing in size until it reaches an upper size limit. Under the assumption that the proportion of late stage cases follows a Bernoulli distribution, each window is evaluated as a possible cluster, and the window with the highest maximum likelihood of being a cluster is assigned a *p* value, which is adjusted for multiple testing. In other words, the scan statistic provides a measure of how unlikely it would be to encounter the observed excess or deficiency of cases in a larger comparison region.

## 9. Concluding Thoughts

I look back at my time at Stanford with much fondness and melancholy. A post-doctoral position can grant a lot of freedom as the burden of writing a dissertation is behind you, but you are not yet overwhelmed with the duties and expectations of a freshly appointed faculty member. It should be a time of discovery and exploration, where your intellect is free to wander less-traveled paths while your mind acts as a sponge as you interact with a new academic environment and other bright minds. The success of such an endeavor is, however, largely at the mercy of the postdoc adviser.

The first day I arrived on campus, André took me for lunch at the Stanford Faculty Club. This was my first encounter with green asparagus, as Europeans typically grow these vegetables covered in a thick layer of mulch and dark plastic so that no sunlight reaches the spears and they remain white. According to André this was a prime example of American practicality; why hassle with growing asparagus in the dark if the green ones taste as good? While disserting on the merits of white versus green asparagus, André pointed to a few Nobel Prize winners assembled around the grazing table. At that moment I first glimpsed the stimulating multi-levels environment I was ready to enter.

Both André and I had the good fortune of receiving our education in a Jesuit institution, and those twelve years of rigorous and challenging curriculum marked us, as we discussed this commonality. One side effect was the feeling of guilt when not working or pushing our limits, a characteristic of over-achiever personalities. I also remember my teachers stating that their goal was to teach us how to learn, not simply fill our heads with academic material. André had a similar philosophy in the sense that beyond what we learned in his classroom, his main contribution was to improve our writing and presentation skills, which turned out to be invaluable assets down the road.

Regarding the application of geostatistics in the medical field, its implementation nowadays remains mainly within a model-based framework (e.g., Diggle and Giorgi 2019), likely because spatial epidemiology is taught primarily in departments of statistics where Matheronian geostatistics is still viewed as a heresy. In my 2009 review paper (Goovaerts 2009b), I wrote “*Critical components to the success of health geostatistics include the publication of applied studies illustrating the merits of geostatistics over spatial statistical methods commonly used in health departments and cancer registries, training through short courses and updating of existing curriculum, as well as the development of user-friendly software*”. This statement is still true eleven years later.
